# Visual closed dumbbell-mediated isothermal amplification (CDA) for on-site detection of *Rickettsia raoultii*

**DOI:** 10.1371/journal.pntd.0010747

**Published:** 2022-09-09

**Authors:** Zheng Gui, Hao Cai, Lin Wu, Qing Miao, Jing feng Yu, Ting Cai, Rui Mao

**Affiliations:** 1 Key Laboratory of Diagnosis and Treatment of Digestive System Tumors of Zhejiang Province, Hwa Mei Hospital, University of Chinese Academy of Sciences, Ningbo, China; 2 Ningbo Institute of Life and Health Industry, University of Chinese Academy of Sciences, Ningbo, China; 3 Graduate School, Inner Mongolia Medical University, Hohhot, Inner Mongolia, China; 4 Department of Parasitology, Inner Mongolia Medical University, Hohhot, Inner Mongolia, China; Galveston National Laboratory, University of Texas Medical Branch, UNITED STATES

## Abstract

Spotted fever group (SFG) rickettsioses are important zoonoses, threatening human health seriously and gradually attracting more attention in the world. SFG rickettsiae are classified as neglected pathogens. If these pathogens are detected at all, they are usually recognized very late in the infection through indirect detection of specific antibodies. Previous studies have shown that *Rickettsia raoultii* (*R*. *raoultii*), a member of the SFG rickettsiae, occurs with increasing incidence in remote countries. Therefore, a rapid detection method for *R*. *raoultii* is in urgently need. In this study, a *R*. *raoultii* diagnosis method by closed dumbbell-mediated isothermal amplification (R-CDA) assay targeting a conserved sequence of the outer membrane protein A (*OmpA*) gene with high sensitivity and specificity was developed. This assay offered a rapid and simple method for on-site detection of *R*. *raoultii*. Firstly, four pairs of R-CDA primers were designed and the optimum primer set was selected to amplify target gene specifically and effectively. Then, a pair of outer primer was designed to accelerate the reaction based on the inner primers to establish the RO-CDA reaction. In addition, the results of real-time amplification curves, melting curves and end-point colorimetric judgements showed that the established visual RO-CDA reaction could accurately detect *R*. *raoultii* without cross-reaction with other closely related pathogens. Furthermore, the detection limit of visual RO-CDA assay was 10 copies/μL, which was feasible for on-site detection with merits of easy-operation, rapidity, high sensitivity, and specificity. In conclusion, the developed RO-CDA detection method could be helpful for pathogen screening and epidemic prevention at the point of care.

## Author summary

Spotted fever group (SFG) rickettsioses are important neglected zoonoses throughout the world, with expanding known distribution, among which *R*. *raoultii* is an emerging member. As the clinical symptoms of *R*. *raoultii* is similar to other diseases, the accurate diagnosis of the pathogen based on the detection of genome plays a vital role in effective treatment and control at low infection levels. Due to the lack of on-site detection technology of *Rickettsia raoultii* in remote areas, the pathogen can only be treated in the late infection stage with specific clinical symptoms. Closed dumbbell-mediated isothermal amplification (CDA) is a method that rapidly amplifies nucleic acids under isothermal condition, which can help to resolve the problem of point of care detection for *R*. *raoultii*. The established *R*. *raoultii* detection approach based on CDA was feasible for on-site detection with merits of easy-operation, rapidity, high sensitivity, and specificity. At last, compared with PCR and LAMP, CDA method is simpler and more efficient to detect *R*. *raoultii*, which helps to improve the efficiency of detection and strengthen the prevention and control of this neglected but widely distributed disease.

## Introduction

Spotted fever group (SFG) rickettsioses are important neglected zoonoses throughout the world, with expanding known distribution. Also, SFG rickettsiae are transmitted by ticks while lice and fleas are vectors for typhus group rickettsiae [1–4]. Currently, the reported SFG rickettsioses cases in China are *Rickettsia heilongjiangensis*, *Rickettsia raoultii*, *Rickettsia slovaca*, *Rickettsia sibirica* and *Rickettsia massiliae* [5–7]. Patients infected with SFG rickettsioses were mainly characterized by fever, chills, headache, dizziness, fatigue, myalgia, and enlarged lymph nodes in the neck, groin and axilla and other symptoms [8–10]. In general, suitable antibiotic treatment would be helpful to tackle this disease effectively at low infection levels. Among them, *R*. *raoultii* is frequently detected in various ticks, especially in *Dermacentor nuttalli* [11,12]. Furthermore, human infection cases by *R*. *raoultii* have been reported continuously in recent years [9,13,14]. Therefore, rapid on-site diagnosis approaches for rickettsioses would be important to determine an appropriate therapeutic strategy [15,16].

Typically, diagnosis of SFG rickettsioses mainly depends on etiological, serological and molecular based approaches. Particularly, molecular biology technologies have attracted much more attention for the merits of accurate, fast and easy-operation [17,18]. With advantages of high sensitivity, specificity and the nucleic acid-based molecular diagnosis, molecular biology-based methods have become the preferred option for the accurate detection of pathogens gradually [19–21]. As well-established nucleic acid analysis approaches, polymerase chain reaction (PCR) and isothermal nucleic acid amplification have become the mainstream for the development of pathogen detection methods [22–24].

Isothermal amplifications are nucleic acid detection methods that amplify nucleic acids under a specific constant temperature other than sophisticated program temperature control in PCR. Moreover, isothermal amplification-based methods reduce instrument requirements and shorten reaction time. Final results are obtained by visual methods, enabling on-site rapid detection [25]. Therefore, isothermal amplification assays showed great potential in scientific and clinical research [26]. Loop-mediated isothermal amplification (LAMP) is one of the well-recognized isothermal amplification techniques applied in clinical diagnosis [27,28]. But LAMP needs specific design of 2 to 3 pairs of primers and the processes of primer design and selection are tedious [29,30]. In order to overcome the shortcomings of LAMP, a novel closed dumbbell-mediated isothermal amplification (CDA) technique was developed [31]. This method takes the advantages of simple primer design, short primer length and low requirement for target sequence, leading to cost-saving for the developed assays. In addition, as far as our knowledge, rapid detection of *R*. *raoultii* by CDA method has not been reported.

In this study, the newly established R-CDA method was developed for *R*. *raoultii* detection in clinical samples. The outer membrane protein A (*ompA*) gene was selected as target which is considered conserved among *R*. *raoultii* to ensure high specificity. The results indicate that the R-CDA method significantly improves the efficiency of field detection of *R*. *raoultii* with high sensitivity and specificity (both 100%) with total 416 tests. We successfully developed a real-time fluorescence and visible on-site CDA assay, which offers an option for control of sudden breakout of *R*. *raoultii* and help to facilitate appropriate treatment of these zoonoses.

## Materials and methods

### Materials

*Rickettsia raoultii* and *Rickettsia sibirica* were extracted from *Dermacentor nuttalli* which were collected by the pathogenic biology laboratory of Inner Mongolia Medical University. They identified the *Rickettsia spp* carried by 74 *Dermacentor nuttalli* individuals in Hulun Buir City of Inner Mongolia in August 2019. DL 2000Marker and dNTPs were purchased from Sangon Biotech (Sangon, Shanghai, China). *Bst* 2.0 WarmStart DNA polymerase and 10 × isothermal amplification buffer (including 200 mM Tris-HCl, 100 mM KCl, 100 mM (NH_4_)_2_SO_4_, 20 mM MgSO_4_, and 1% Triton X-100) were purchased from New England BioLabs (Ipswich, MA, USA). EVA Green and GelRed were obtained from Biotium (Hayward, CA, USA). Primers were provided by BGI Biological Engineering Technology and Services Co. Ltd (Shenzhen, China). Other reagents, unless specified, were obtained from Sigma-Aldrich (St. Louis, MO, USA).

### R-CDA primer design

**Fig 1:**
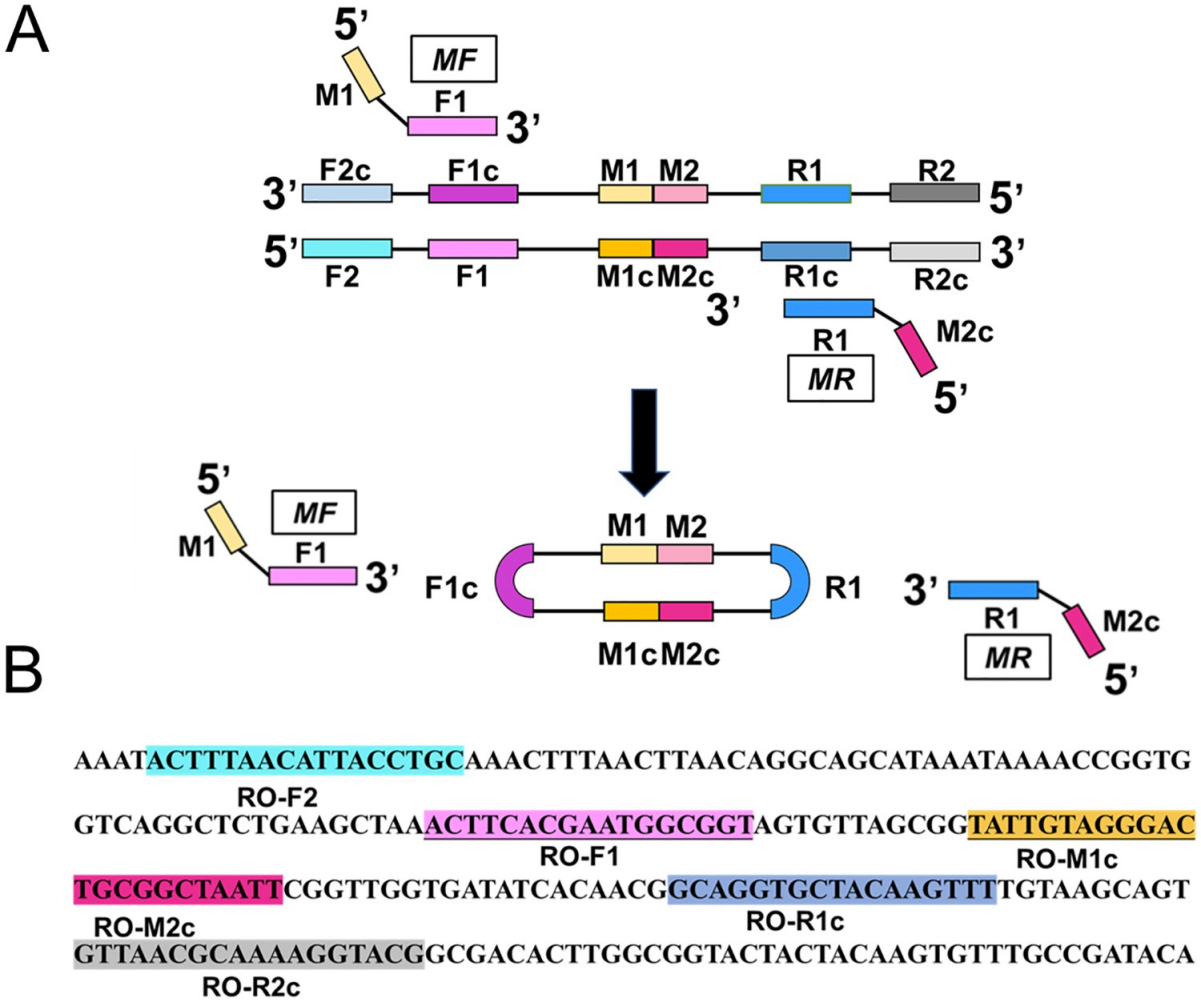
Principle of closed dumbbell-mediated isothermal amplification (CDA) method. (A) Overall scheme for primers used in CDA method. (B) Partial sequence of the *ompA* gene of *R*. *raoultii* used for designing the primers of CDA method.

In this study, a *R*. *raoultii* diagnosis method by closed dumbbell-mediated isothermal amplification assay called R-CDA assay was developed. Four pairs of R-CDA primers were designed by DNAMAN Version 8.0 software according to the *R*. *raoultii ompA* gene. R-CDA primers (typically 18–24 nts in length) can be obtained directly from the *ompA* gene sequence (Table 1). The scheme designed by R-CDA primers is shown in Fig 1A. The sequences of F1, R1 and M represent "forward 1", "reverse 1" and "middle" respectively. The site with the lowercase "c" stands for "complementary". The internal sequence between F1c and R1 is M, which is divided into M1 and M2 on average. Under this structure, MF contains a sequence complementary to M1 (M1c) and a complementary sequence to F1c (F1). MR contains sequences (M2) that complement to M2c and R1. In order to further optimize the developed R-CDA assay, outer primers (F2 and R2) were designed to accelerate the R-CDA reaction (Table 2). The locations of all R-CDA primers used in this study were marked with different colors in Fig 1B. The primer sequences are listed in Tables 1 and 2 and synthesized by BGI Biological Engineering Technology and Services Co. Ltd (Shenzhen, China).

**Table 1:** Screening of R-CDA basic primer sequences for the mapping genes of *Rickettsia raoultii*.

Target	Method	Primer	Sequence (5´→3´)
*ompA*	CDA	R-MF-1	GTCCCTACAACA-ACTTCACGAATGGCGGT
		R-MR-1	TGCGGCTAATT-AAACTTGTAGCACCTGC
		R-MF-2	TCCCTACAACAACTTCACGAATGGCGGT
		R-MR-2	TGCGGCTAATTCAAAACTTGTAGCACCT
		R-MF-3	TCCCTACAACACTTCACGAATGGCGGTAG
		R-MR-3	TGCGGCTAATTTACAAAACTTGTAGCAC
		R-MF-4	TCCCTACAACAAAACTTCACGAATGGCGG
		R-MR-4	TGCGGCTAATTTACAAAACTTGTAGCAC

**Table 2:** Primer sequences of RO-CDA and PCR targeting at map gene of *Rickettsia raoultii*.

Target	Method	Primer	Sequence (5´→3´)
*ompA*	CDA	R-MF	GTCCCTACAACA-ACTTCACGAATGGCGGT
		R-MR	TGCGGCTAATT-AAACTTGTAGCACCTGC
		R-F2	ACTTTAACTTAACAGGCAGC
		R-R2	CGTACCTTTTGCGTTAAC
	PCR	R-F	ACTAGGTGCGAATAGACCC
		R-R	CGTACCTTTTGCGTTAACACT

### R-CDA reaction and optimization

The synthesized *R*. *raoultii* gene fragment was used as positive control template. Four pairs of R-CDA primers were designed and adopted to amplify the same target by R-CDA reaction mixture respectively. A total of 25 μL of each R-CDA reaction was adopted, including 1 μL *Bst* DNA polymerase, 12.5 μL 10 × isothermal amplification buffer, 1 μL MF, 1 μL MR, 1 μL nucleic acid target, 1 μL for each Eva green and HNB, an appropriate amount of nucleic acid templates and RNase-free water was used to adjust the volume to 25 μL. Into this system, the outer primers were added 2 μL F2 and 2 μL R2, which considered as RO-CDA. To avoid aerosol pollution generated in the assays, 30 μL paraffin oil was added before amplification. The amplification reaction was generally carried out at 60°C to 65°C for 60 min and terminated by heating 10 min at 85°C in real time PCR (Sansure biotechnology, Changsha, China). In the experiment, positive control consisted of DNA of *R*. *raoultii* from field-collected ticks and the negative control was nuclease-free water. Due to the insoluble magnesium pyrophosphate produced by RO-CDA amplification, hydroxy naphthol blue (HNB) was added to indicate the reduction of Mg^2+^ in the above endpoint monitoring. The purple is negative, and the light blue is positive for endpoint judgement of amplification results. Lastly, to optimize the reaction system for *R*. *raoultii* detection, different temperatures at 60°C, 61°C, 62°C, 63°C, 64°C and 65°C were tested.

### Sensitivity and specificity of RO-CDA assay

Outer primers were added to the original primers to achieve a more efficient CDA reaction, called RO-CDA. Based on determining the optimum temperature, the *R*. *raoultii* DNA ranging from 1.0×10^6^ to 1 copies/μL were diluted gradiently by sterile water to evaluate the sensitivity of the developed RO-CDA method. As to specificity determination, the closely related *Rickettsia sibirica* and *Anaplasma ovis*, the distantly related *Klebsiella pneumoniae* and *Pseudomonas aeruginosa* were tested for cross-reactivity in this assay. DNA samples of *Anaplasma ovis* were extracted from *Dermacentor nuttalli* which were provided by the pathogenic biology laboratory of Inner Mongolia Medical University. They identified *Anaplasma ovis* by amplifying the msp4 gene of Anaplasma in Hulun Buir City of Inner Mongolia. DNA samples of *Klebsiella pneumoniae* and *Pseudomonas aeruginosa* were cultured and extracted from the sputa of patients. DNA samples of *Escherichia coli* (CGMCC 1.12883), *Shigella* (CVCC 1597), *Staphylococcus aureus* (CGMCC 1.6750), *Vibrio parahaemolyticus* (CGMCC 1.1997), *Listeria monocytogenes* (CGMCC 1.9144) and *Streptococcus agalactiae* (CICC 10465) were extracted using DNA purification kits. The experimental results were obtained by amplification curves, melting curves and endpoint color.

### *Rickettsia raoultii* PCR amplification and sequencing

The extracted *R*. *raoultii* samples were amplified by PCR (Forward primer: ACTAGGTGCGAATAGACCC, Reverse primer: CGTACCTTTTGCGTTAACACT). The PCR reaction was performed in 40 μL volume, including 20 μL Taq PCR Master Mix, 1 μL for each primer (40 μM), 2 μL for target, and nuclease-free water was used to adjust the volume to 40 μL. PCR reaction running procedures: denaturation at 95°C for 30 s, annealing at 56°C for 30 s and extension at 72°C for 30 s, for a total of 35 cycles. The PCR products were analyzed by agarose gel electrophoresis and sequenced by Sangon setting as standard method.

## Results

### Confirming and optimizing of RO-CDA primer for *Rickettsia raoultii*

**Fig 2:**
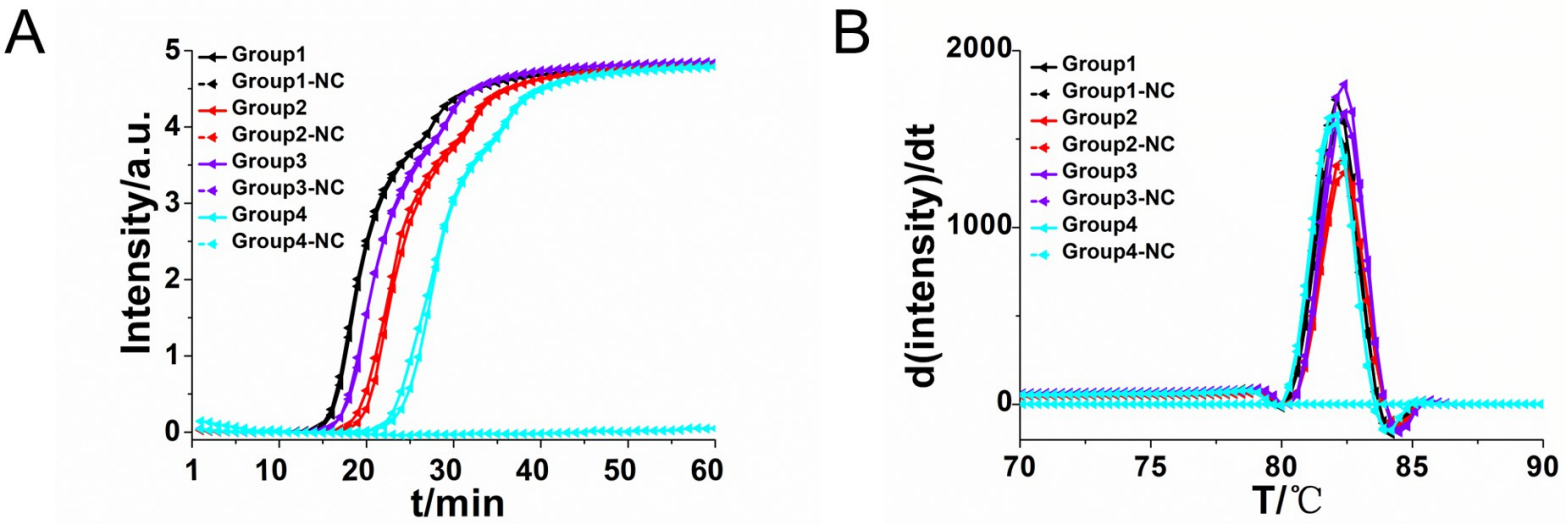
Amplifications of *R*. *raoultii* DNA by R-CDA monitored by real-time PCR carried out at 60°C for 60 min. (A) Real-time R-CDA of different groups for *R*. *raoultii* DNA (each set 2 positive reactions and 2 negative reactions). (B) Melting curve analysis of R-CDA products by real-time PCR. NC, negative control reaction without the template.

**Fig 3:**
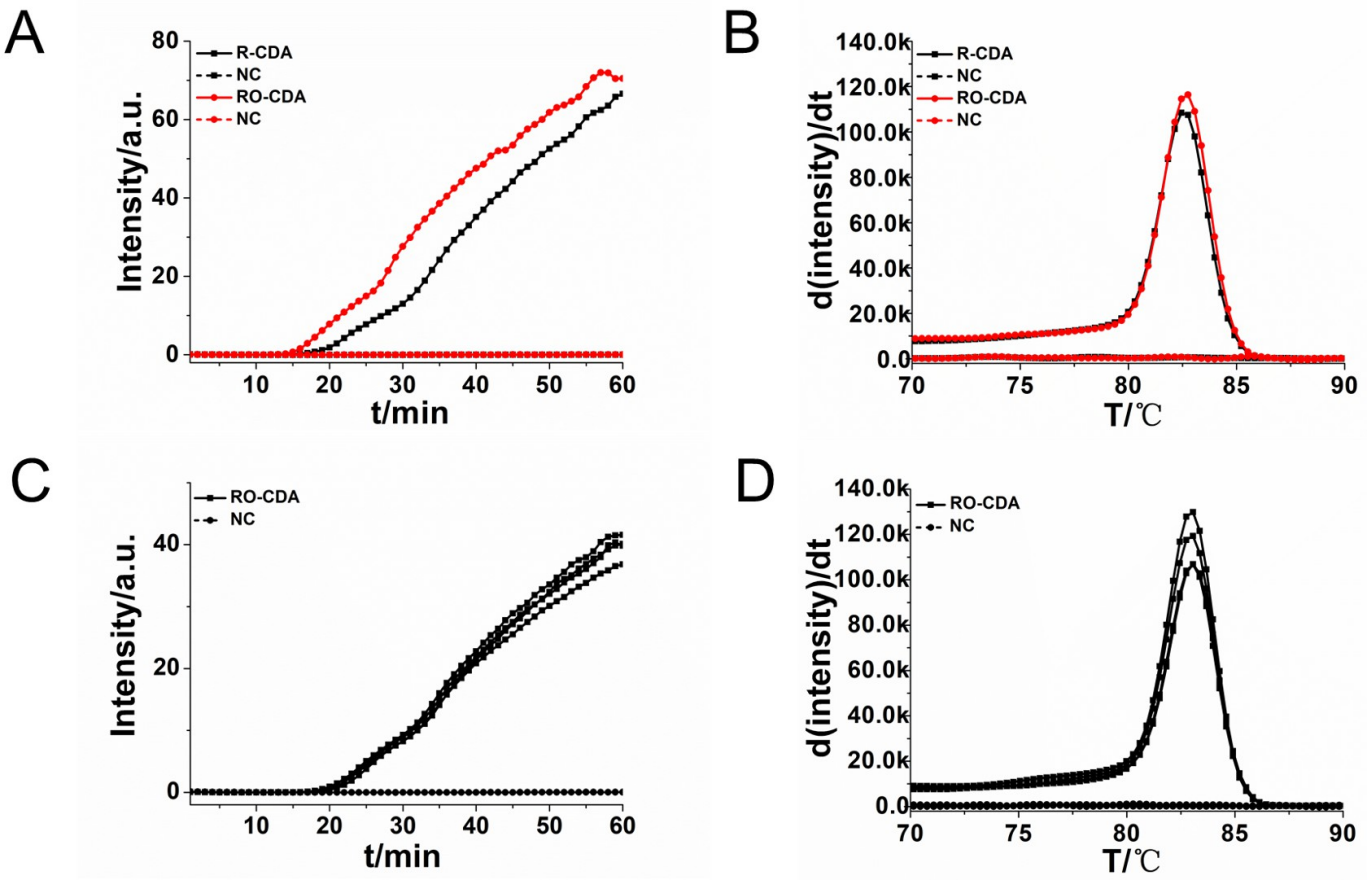
Amplifications of *R*. *raoultii* DNA by R-CDA and RO-CDA monitored by real-time PCR. (A) Amplification plot of real-time R-CDA and RO-CDA for *R*. *raoultii* DNA. (B) Melting curve analysis of CDA and RO-CDA products. (C) Repeatability analysis of the RO-CDA monitored by real- time PCR at 60°C. (D) Repeatability analysis of melting curve analysis of RO-CDA products. PC, positive reaction with the template; NC, negative control reaction without the template.

Four pairs R-CDA primers targeted at *R*. *raoultii ompA* gene were tested to obtain the optimum primer set. The cycle threshold of R-CDA reaction was negatively correlated with the amplification efficiency. As shown in Fig 2, the amplification curve of R-CDA method showed that the first group of primers were the best (Table 1). The threshold detection time of the 10^6^ copies of *ompA* gene was 15 min, the melting temperature of the amplified product was 82.5°C and no non-specific amplification was observed in negative control. Both the real-time amplification curve and the melting curve showed that the CDA method for *R*. *raoultii* detection exhibited good repeatability and stability. And then, outer primers were added to the original primers to achieve a more efficient CDA reaction, called RO-CDA. As shown in Fig 3A, the threshold time of detecting *Rickettsia* (1.0 × 10^6^ copies) was shortened 3 min by RO-CDA. The reaction efficiency of CDA method was improved by adding outer primers. After screening of outer primers, the reaction temperature of *R*. *raoultii* RO-CDA detection was optimized after incubation at 60°C, 61°C, 62°C, 63°C, 64°C and 65°C, respectively. The optimal reaction temperature for the developed RO-CDA method was set as 60°C to ensure positive detection with a low DNA concentration (S1 Fig).

To further evaluate the repeatability of the developed assay, 10^7^ copies of the *R*. *raoultii* DNA was amplified in both four positive and negative replicates independently by RO-CDA. Both the real-time amplification curve (Fig 3C) and melting curve (Fig 3D) indicated a good repeatability of the RO-CDA method. The melting curves in Fig 3B and 3D were exactly the same, indicating the outer primers only stimulate the acceleration of the amplification reaction, without non-specific amplification. All these optimized reactions were utilized in further experiments.

### Sensitivity and specificity of RO-CDA

**Fig 4:**
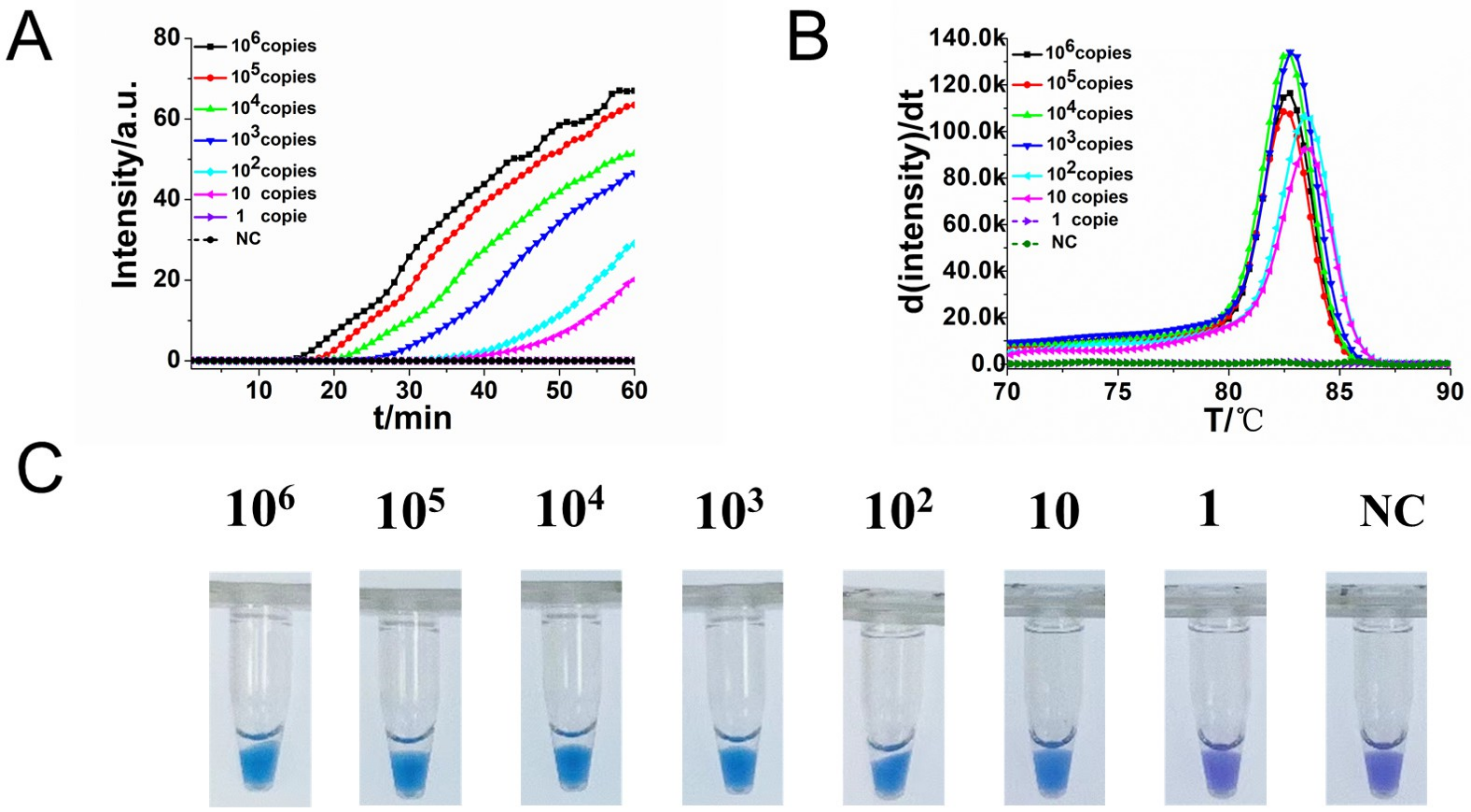
Sensitivity analysis of the *R*. *raoultii* RO-CDA method by real-time and visual approaches. (A) RO-CDA amplification monitored by real-time PCR every 1 min at different concentrations of DNA. Reaction was performed at 60°C for 60 min. (B) Melting curve analysis of RO-CDA products at different concentrations of DNA. (C) Sensitivity analysis of *R*. *raoultii* detection by visually RO-CDA. The DNA concentrations were as follows: 10^6^ copies/μL, 10^5^ copies/μL, 10^4^ copies/μL, 10^3^ copies/μL, 10^2^ copies/μL, 10 copies/μL, 1 copy/μL and negative control (nuclease-free water). NC, negative control reaction without the template.

**Fig 5:**
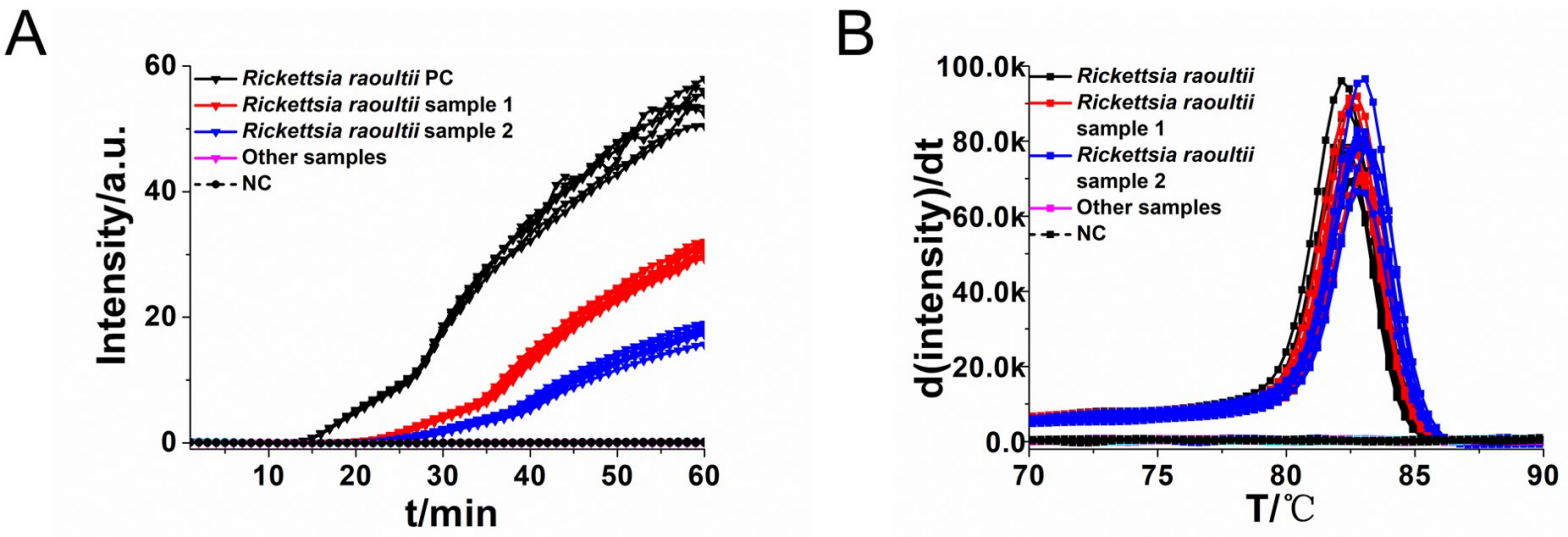
Real time RO-CDA assay. Real-time RO-CDA reactions were carried out at 60°C for 60 min. (A) Real-time RO-CDA for *R*. *raoultii* DNA positive controls (*Rickettsia* PC), different *R*. *raoultii* samples (HLBE 407 and 408), other rickettsial and non rickettsial samples, (*Rickettsia sibirica*, *Anaplasma ovis*, *Klebsiella pneumoniae*, *Pseudomonas aeruginosa*, *Escherichia coli*, *Shigella*, *Staphylococcus aureus*, *Vibrio parahaemolyticus*, *Listeria monocytogenes* and *Streptococcus agalactiae*) and no template control (NC). (B) Melting curve analysis of the RO-CDA products by real-time PCR. PC, positive reaction with the template; NC, negative control reaction without the template.

**Fig 6:**
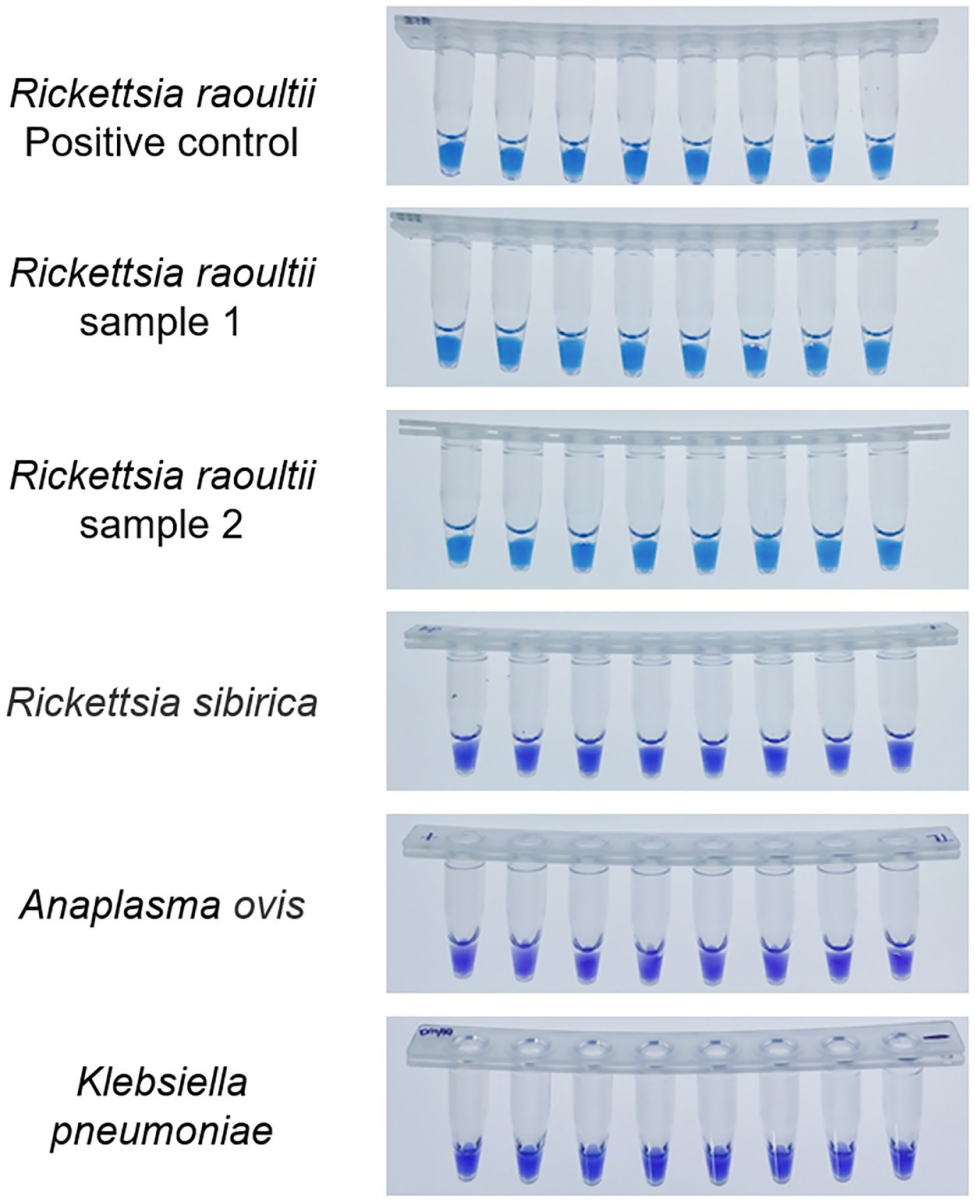
Colorimetric RO-CDA assay using HNB. Line 1, positive controls (10^6^ copies of templates); Line 2 and Line 3, samples extracted from *R*. *raoultii*; Line 4, samples extracted from *Rickettsia sibirica*; Line 5, samples extracted from *Anaplasma ovis*; Line 6, samples extracted from *Klebsiella pneumoniae*. PC, positive reaction with the template. Due to the limited space of the manuscript, other samples are not displayed.

To determine the sensitivity of RO-CDA method, the DNA concentration of 10-fold diluted *R*. *raoultii* DNA from 10^6^ copies/μL to 1 copy/μL were applied. The amplification curve and the color change of the reaction products showed that the lowest detection limit of RO-CDA method for *R*. *raoultii* was 10 copies/μL (Fig 4A and 4C). In addition, the melting curve analysis of *R*. *raoultii* detected by real-time fluorescence RO-CDA showed that the Tm values (84°C) of 10^2^ and 10 copies were slightly different which would be affected by high concentration of non-targeted DNA sequences (Fig 4B). Regarding to the specificity of RO-CDA, the RO-CDA primers specifically amplified the *ompA* sequence of *R*. *raoultii* (Fig 5A). Melting curve analysis showed that the Tm value was 82.5°C (Fig 5B), and there was no difference between the positive control and different DNA samples. In addition, *Dermacentor nuttalli* samples from different regions were adopted to evaluate the repeatability, practicability, and reliability of the developed RO-CDA method. The visible endpoint results are shown in Fig 6.

### Analysis of sequencing results

The extracted *R*. *raoultii* DNA from different *Dermacentor nuttalli* individuals were amplified by PCR targeting at *ompA* gene, and the products were sequenced and analyzed. Based on the representative data of *R*. *raoultii*, the sequence alignment results illustrate 99.41% homology among *R*. *raoultii* sequences (S2 and S3 Figs) used Basic Local Alignment Search Tool (BLAST) in the National Center for Biotechnology Information (NCBI). In addition, the *ompA* gene phylogenetic tree of *Rickettsia spp*. revealed that *R*. *raoultii* was the predominant found in Mongolian *Dermacentor nuttalli*, and the genetic distances with different *Rickettsia* species were 0.10 (S4 Fig).

## Discussion

Nucleic acids-based assays, such as real-time PCR, has been effectively applied for the detection of *Rickettsiae* [32]. However, these methods are not yet widely used in rural hospitals [33]. It can be speculated that *R*. *raoultii* may be misdiagnosed due to the lack of timely detection, especially for residents who live in remote areas with high risk of tick exposure. Thus, the main weakness in disease diagnosis is that people in rural areas have no access to modern equipment to achieve accurate and rapid pathogen identification, which may hinder effective treatment of *Rickettsia* [15]. In this study, a convenient and efficient detection method of *R*. *raoultii* was developed to facilitate appropriate treatment of this zoonosis.

Since the recognition of the well-established isothermal amplification method, LAMP technology has been applied to detect a variety of pathogenic microorganisms, such as *Anaplasma*, *Ehrlichia*, *SARS-CoV* [34–36]. It is considered that LAMP-based diagnosis methods have the advantage of being rapid, simple, and accurate, but are restricted by the complexity primer design and non-specific amplification [37]. Here, compared with LAMP, CDA method was simpler and more efficient to detect *R*. *raoultii*. So far, there was no CDA assay reported for the detection of vector-borne pathogens. This study has successfully established and optimized the method of CDA amplification of *R*. *raoultii*.

For developing the CDA-based detection, two primers were designed that amplify the target gene (10^6^ copies/μL) within one hour under the isothermal condition of 60°C. Visual detection of *R*. *raoultii* by CDA also showed good repeatability. The specificity test revealed that the CDA assay did not identify the closely related *Rickettsia sibirica* and *Anaplasma ovis*, and there was no cross-reaction with *Klebsiella pneumoniae*, *Pseudomonas aeruginosa*, *Escherichia coli*, *Shigella*, *Staphylococcus aureus*, *Vibrio parahaemolyticus*, *Listeria monocytogenes* and *Streptococcus agalactiae*. The sequences of partial samples of *R*. *raoultii* were compared with the sequences on BLAST, and the homology was 99.41%. Overall, the reliability, specificity and repeatability of the developed CDA method is very good.

CDA assay is generally more convenient than most of the currently used methods for detection of *Rickettsia spp*., i.e. LAMP and others because of simpler primer design [16,38]. LAMP method and recombinase polymerase amplification (RPA) assay are based on a complex primer design, which complicates the detection of rickettsiae. Besides, Hanaoka et. al found that the limit of detection for *rickettsiae* was 100 copies/μL by LAMP method [39], while the detection limit of CDA assay was 10 copies/μL. Furthermore, the sensitivity and specificity were both 100% from total 416 tests by CDA assay, which were superior to LAMP (Table 3). Yong Qi et. al developed a RPA-based assay for detection of *Rickettsi*a [40], but the complex reaction mixtures and expensive recombinase polymerase could limit further application. Moreover, the development CDA assay saves tedious equipment operation, reduces the requirements for staff, and is more suitable for grass-roots units and epidemic scenes to carry out pathogen detection. The colorimetric detection system of the assay impresses with the speed with which results are obtained. It requires only mixing the extracted DNA with the reaction mixture and incubation at 60°C for one hour. Furthermore, the colorimetric CDA assay has the potential to become a tool for on-site monitoring of *R*. *raoultii* in arthropods.

**Table 3:** Determination of sensitivity and specificity of RO-CDA assay for *Rickettsia raoultii*.

Species	Sample numbers		Sensitivity	Specificity	Accuracy
	RO-CDA	defined	95% CI *	95% CI *	95% CI *
*Rickettsia raoultii*	96	96	1.0 (96.2–100.0)	1.0 (98.8–100.0)	1.0 (99.1–100.0)
*Rickettsia sibirica*	0	32			
*Anaplasma ovis*	0	32			
*Klebsiella pneumoniae*	0	32			
*Pseudomonas aeruginosa*	0	32			
*Escherichia coli*	0	32			
*Shigella*	0	32			
*Staphylococcus aureus*	0	32			
*Vibrio parahaemolyticus*	0	32			
*Listeria monocytogenes*	0	32			
*Streptococcus agalactiae*	0	32			
Total	96	416			

* CI: confidence interval.

Statistical analysis was carried out by online program of “Diagnostic test evaluation calculator”, https://www.medcalc.org/calc/diagnostic_test.php.

However, SFG rickettsiae has a wide range of species and no CDA assay for detection of other *Rickettsia* species have been found. In the future, CDA method should be developed for identifying a class of *Rickettsia* and distinguishing specific *Rickettsia* species by a microfluidic chip to achieve multiplex on-site diagnosis as an approach to precise medicine in rural areas [41].

## Conclusion

In this study, a sensitive and specific CDA method for visual detection of *R*. *raoultii* was established, which could be applied to the field detection of *R*. *raoultii* in remote areas. The *R*. *raoultii* CDA method may help to improve the efficiency of detection and strengthen the prevention and control of this neglected but widely distributed disease.

## Supporting information

S1 Fig Amplification plot of real time RO-CDA for *Rickettsia raoultii* at different temperatures. Each set 4 positive reactions and 4 negative reactions.
(TIF)

S2 Fig Agarose gel electrophoresis of PCR products of the amplified part of the *ompA* gene of *Rickettsia raoultii*. M: DL 2000 DNA Marker; NC: negative control.
(TIF)

S3 Fig Sequences alignment of partial samples of *Rickettsia raoultii* by DNAMAN. The sequence alignment results in GenBank: i.e. accession no: AH015610.2, CP010969.1, AH009131.2, KM288513.1, KM288500.1, KM288495.1, MK304548.1, HQ232221.1, OL348252.1, JX683119.1.
(TIF)

S4 Fig Phylogenetic tree based on the *ompA* gene of *Rickettsia raoultii*. HLBE: Hulun Buir City of Inner Mongolia, China.
(TIF)
