# Supplementary figures and images for: Visual closed dumbbell-mediated isothermal amplification (CDA) for on-site detection of Rickettsia raoultii
Source: PLoS Negl Trop Dis. 2022 Sep 9;16(9):e0010747. doi: 10.1371/journal.pntd.0010747 (PMC9491570; doi:10.1371/journal.pntd.0010747)

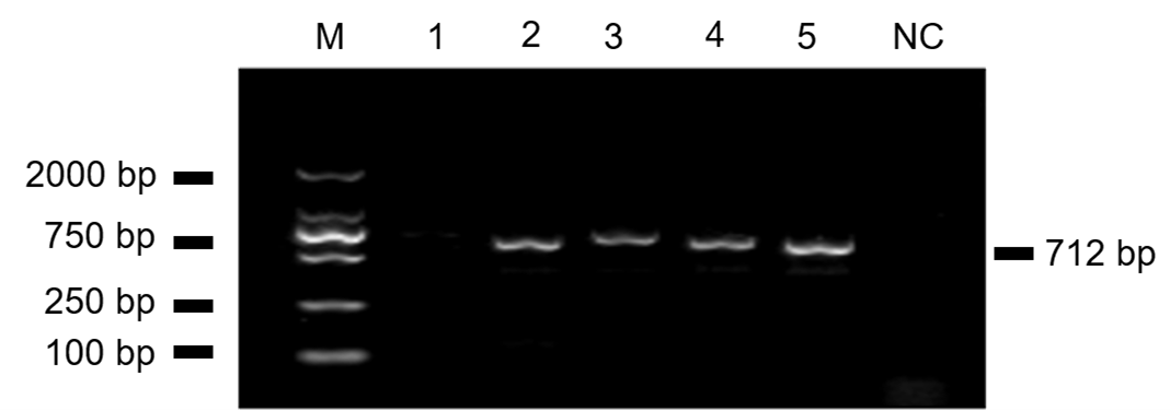

Supplement: S2 Fig — M: DL 2000 DNA Marker; NC: negative control. (TIF) [file pntd.0010747.s002.tif]

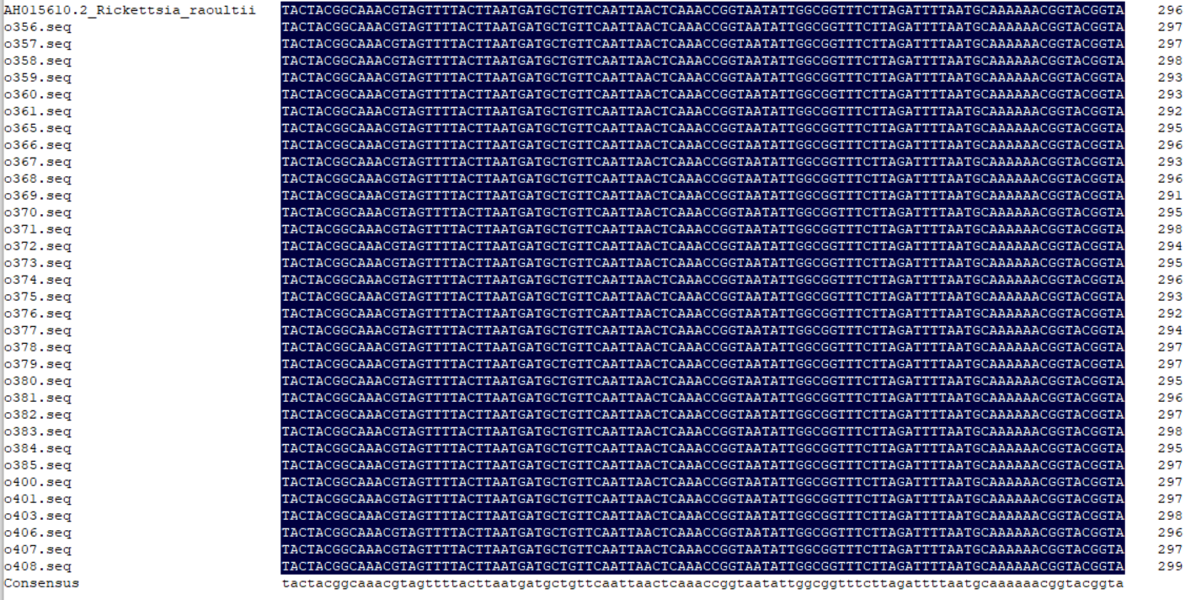

Supplement: S3 Fig — The sequence alignment results in GenBank: i.e. accession no: AH015610.2, CP010969.1, AH009131.2, KM288513.1, KM288500.1, KM288495.1, MK304548.1, HQ232221.1, OL348252.1, JX683119.1. (TIF) [file pntd.0010747.s003.tif]

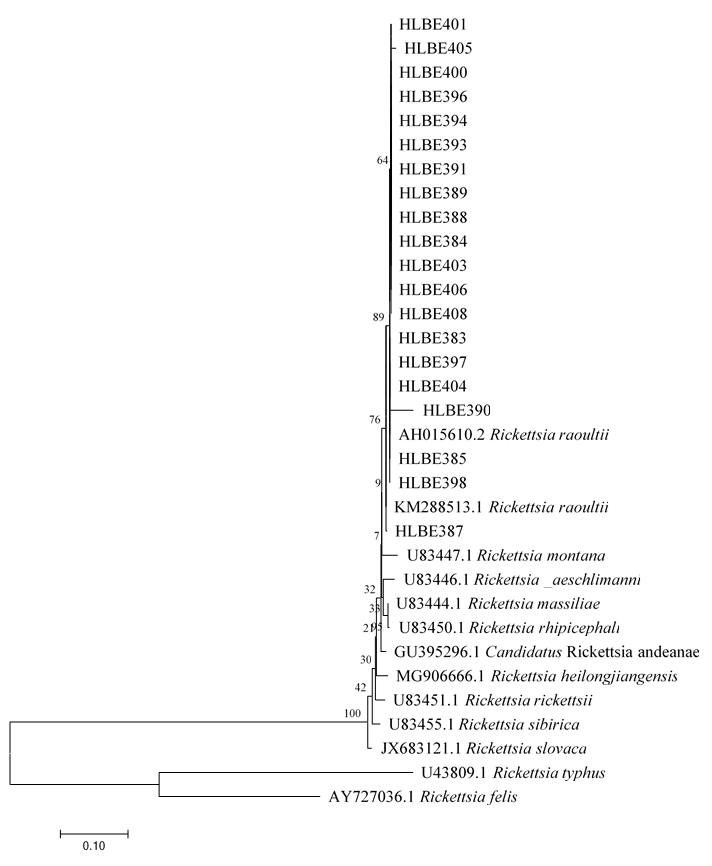

Supplement: S4 Fig — HLBE: Hulun Buir City of Inner Mongolia, China. (TIF) [file pntd.0010747.s004.tif]
